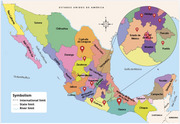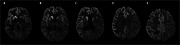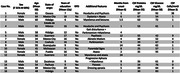# Incidence of Creutzfeldt‐Jakob Disease in a Tertiary Care Referral Center in Mexico City

**DOI:** 10.1002/alz70860_100248

**Published:** 2025-12-23

**Authors:** Ilse Murrieta Hernandez, Enrique Isaac Tetlalmatzi Azuara, Hector Tellez Lucero, Juan Carlos Lopez Hernandez, Enrique Piña Rosales, Brenda Dzul Garcia, Raul Medina Rioja

**Affiliations:** ^1^ Instituto Nacional de Neurología y Neurocirugía “Manuel Velasco Suárez”, Mexico, DF, Mexico; ^2^ Universidad Nacional Autónoma de México, Mexico, DF, Mexico

## Abstract

**Background:**

Creutzfeldt‐Jakob disease (CJD) is a progressive, irreversible, and fatal disease associated with the misfolding of a protein in the central nervous system. Several surveillance programs emerged worldwide after the epidemic of the early 2000s. In Mexico, information about its epidemiology is scarce. A case series published in 2022 analyzed 24 cases over six years; however, its incidence and prevalence remain unknown.

**Method:**

We analyzed patients evaluated in the Emergency Department of the “Manuel Velasco Suárez” National Institute of Neurology and Neurosurgery in Mexico City, whose final diagnosis was sporadic Creutzfeldt‐Jakob disease. Using the electronic medical records, we calculated the incidence of the disease in the Emergency Department and analyzed clinical, imaging, EEG, and cerebrospinal fluid variables. Finally, we identified the states of origin of the analyzed patients.

**Result:**

In 2023, 9,060 patients were treated in our Emergency Department, with 5 cases of CJD detected. In 2024, 9,934 patients were treated, and 10 cases were identified. The incidence of CJD in our Emergency Department was 0.06% in 2023 and 0.10% in 2024. Another patient who met the diagnostic criteria was identified but was treated as an outpatient. In total, 16 cases met the diagnostic criteria. Only 3 patients were originally from Mexico City (Figure 1). Two patients presented with isolated dementia at diagnosis, while clinical presentations ranged from classic cases to atypical manifestations, such as psychosis and primary progressive aphasia. Two patients had a family history of the disease. The average age was 58 years, and 69% were male. All patients had MRI abnormalities (Figure 2). Clinical characteristics are summarized in Table 1.

**Conclusion:**

Creutzfeldt‐Jakob disease is a condition primarily characterized by rapidly progressive dementia, which can present with varied clinical features. It leads to loss of independence in most cases within a year. The current epidemiology of CJD in Mexico is unknown; understanding its incidence could help develop better strategies for managing patients presenting with rapidly progressive dementia in emergency settings.